# Supplementary material for: High-strength and machinable load-bearing integrated electrochemical capacitors based on polymeric solid electrolyte
Source: Nat Commun. 2023 Jan 4;14:64. doi: 10.1038/s41467-022-35737-w (PMC9812976; doi:10.1038/s41467-022-35737-w)
Supplement: Supplementary file 1 — Supplementary Information [file 41467_2022_35737_MOESM1_ESM.pdf]

Supplementary Information

High-strength and machinable load-bearing integrated electrochemical capacitors based on polymeric solid electrolyte

Jinmeng Zhang[1#], Jianlong Yan[1#], Yanan Zhao[1], Qiang Zhou[1], Yinxing Ma[2], Yaxian Zi[1], Anan Zhou[1], Shumin Lin[1], Longhui Liao[1], Xiaolan Hu[1*], Hua Bai[1*]

1 College of Materials, Xiamen University, Xiamen 361005, PR China

2 *College of Chemistry and Chemical Engineering, iChEM, Xiamen University, Xiamen 361005, PR China*

** Corresponding author*

[#] These authors contributed equally to this work

E-mail addresses: baihua@xmu.edu.cn, xlhu@xmu.edu.cn

Table of contents

Supplementary Figures

Supplementary Fig. 1 | DSC measurement of precursors of EP_{100}, EP_{70}, EP_{60}, EP_{50}, and EP_{40}

Supplementary Fig. 2 | DSC test of cured EP_{50}

Supplementary Fig. 3 | Gel time test of solid EP_{100} and EP_{50}

Supplementary Fig. 4 | FT-IR spectra of the uncured mixture of EP_{50} (liquid EP), different EPs

Supplementary Fig. 5 | SEM images of EP_{100}, EP_{70}, EP_{60}, EP_{55}, EP_{50}, EP_{45}, EP_{40}

Supplementary Fig. 6 | Dynamic mechanical analysis (DMA) of different EPs

Supplementary Fig. 7 | Mechanical strength test of different EPs

Supplementary Fig. 8 | Impedance spectra of different solid electrolytes

Supplementary Fig. 9 | Photos of LEID-3 with different curved shapes

Supplementary Fig. 10 | Electrochemical test of Reduction parts of automobile shell

Supplementary Fig. 11 | Specific capacitance of LEID-3

Supplementary Fig. 12 | Ragone plot of LEID-3 based on the areal of the whole device

Supplementary Fig. 13 | Physical map and electrochemical test of different bending deflections

Supplementary Fig. 14 | SEM pictures before and after bending

Supplementary Fig. 15 | Electrochemical performance of the device after 100 cycles

Supplementary Fig. 16 | BET test of the CF

Supplementary Fig. 17 | The photos of LEID-3, LEID-5, LEID-7, LEID-9

Supplementary Fig. 18 | Flexural test of LEIDs with different number of layers

Supplementary Fig. 19 | Electrochemical properties of three-layered subdevice in LEID-7 （I）

Supplementary Fig. 20 | Electrochemical properties of five-layered subdevice in LEID-7 （I）

Supplementary Fig. 21 | Electrochemical properties of seven-layered subdevice in LEID-7 （I）

Supplementary Fig. 22 | Electrochemical properties of LEID-7 （I） in parallel mode

Supplementary Fig. 23 | Mechanical strength test of CM-3, LEID-3, and LEID-7 （II）

Supplementary Fig. 24 | Specific capacitance of LEID-7 （II） and its subdevice in different modes

Supplementary Tables

Supplementary Table 1 | The composition of the precursor mixture for different electrolytes

Supplementary Table 2 | Conductivity comparison of ionic liquids electrolyte and organic electrolytes

Supplementary Table 3 | The Ionic conductivity and mechanical strength comparison of the solid electrolytes in literature with EP_{50} in this work

Supplementary Table 4 | Sample thickness and composition of LEIDs

Supplementary Table 5 | Comparison of the specific capacitance of LEID in this work with other solid-state supercapacitors in the literature

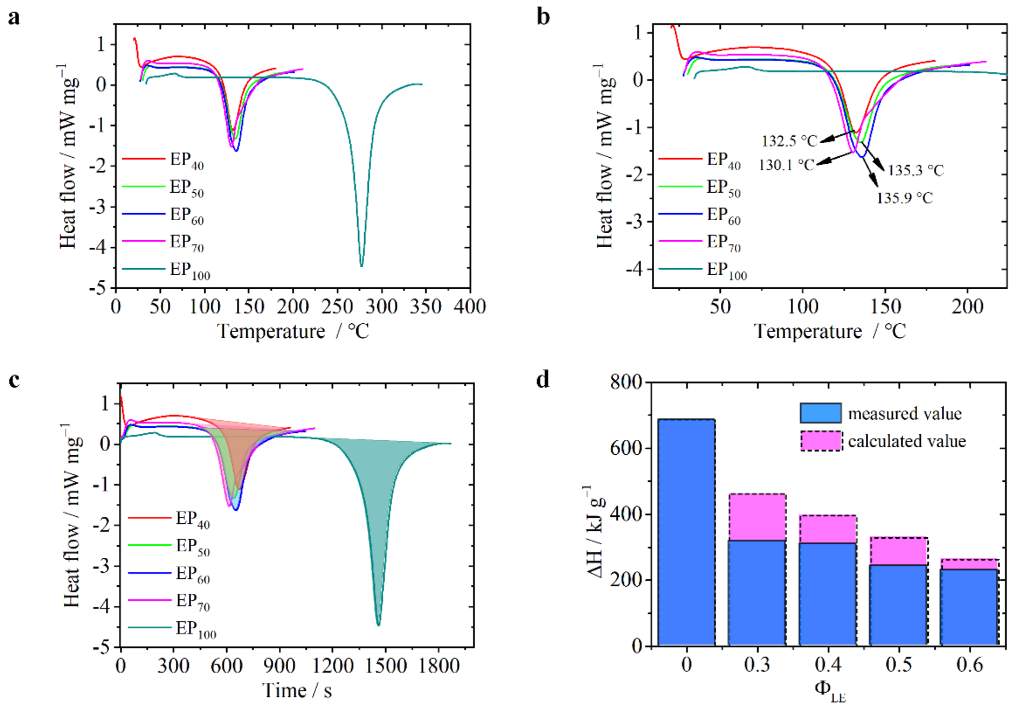

Supplementary Fig. 1 | ** DSC measurement of precursors of EP_{100}, EP_{70}, EP_{60}, EP_{50}, and EP_{40}. **a DSC curves. **b** Low-temperature part of the curves in **(a)**. **c** Mathematical integration plot of the heat flux for the calculation of enthalpy. **d** Enthalpy change (ΔH) of the curing reaction. Calculated values were obtained from the ΔH of pure epoxy resin and the content of epoxy resin in the solid electrolytes (neglecting the change of heat capacity).

As the content of ionic liquid increases, the exothermic peak shifts to lower temperature. Supplementary Fig. 1 shows that the maximum exothermic peak temperature of various samples containing lithium salt is around 133 °C, but the maximum exothermic peak temperature of pure epoxy resin is ~ 275 °C. Therefore, lithium salt has a catalytic effect on the solidification of the polymer electrolytes[1-5].

The enthalpy of the curing reaction was calculated according to the DSC curves, as shown in

Supplementary Fig. 1. The curing enthalpy of pure epoxy resin was 658.7 kJ g^{-1}, and when LE was added, the curing enthalpy of the mixture was expected to decrease. The measured enthalpy was smaller than the calculated value, indicating that the crosslinking density was reduced. With the addition of LE, the concentration of epoxy resin prepolymer decreased, and consequently, the crosslinking probability decreased.

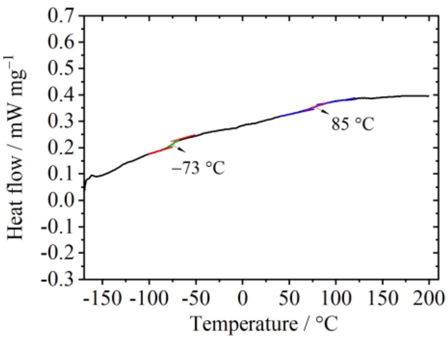

Supplementary Fig. 2 | DSC test of cured EP_{50}.

Two glass transition temperatures at −73 °C and 85 °C were observed on the DSC curve. The first one should be related to the LE phase, and the second one can be ascribed to the epoxy resin phase plasticized by the LE.

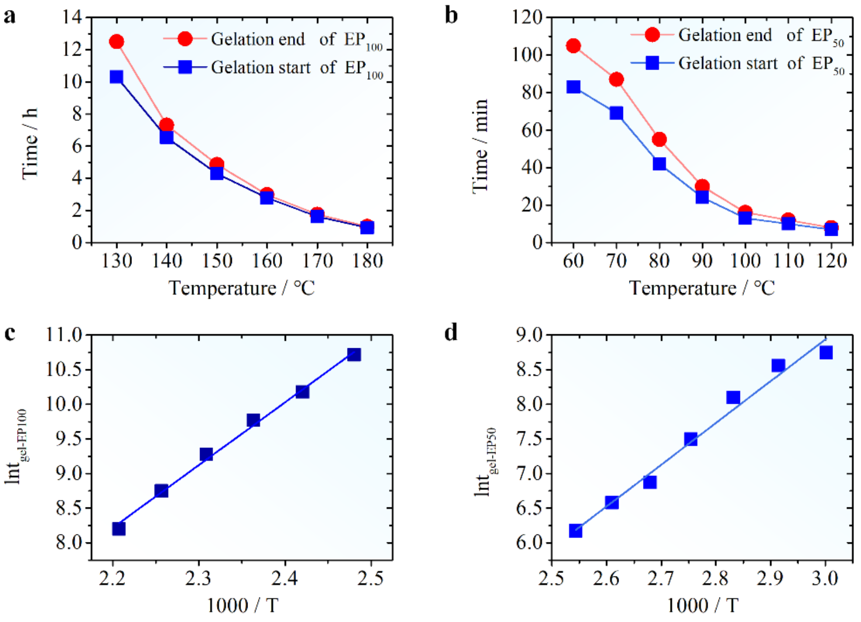

Supplementary Fig. 3 | a Gel temperature-time of EP_{100} at different temperature. **b** Gel temperature-time of EP_{50} at different temperature. **c** Fitted of gel time curves of EP_{100} for the calculation of curing activation energy. d Fitted of gel time curves of EP_{50} for the calculation of curing activation energy.

Supplementary Fig. 3a, b are the gel temperature-time curves of EP_{100} and EP_{50} samples. After introducing the electrolyte, the time for the system to reach a certain degree of crosslinking is shortened. The gel activation energy of the epoxy resin-based polymer electrolyte system can be obtained according to the gel start time at different temperatures. According to the gel model formula $t = Ae^{E/RT}$, there should be a linear relationship between $\ln t_{gel}$ and the T^{-1}, and the slope of the line is *E*/*R*, where E is the curing activation energy and R is the ideal gas constant. As shown in Supplementary Fig. 3c, d, the slope of curve of EP_{100} is obviously higher than that of EP_{50}, and the calculated activation energies of EP_{100} and EP_{100} are 75.2 kJ mol^{-1} and 49.8 kJ mol^{-1}, respectively, indicating that the addition of electrolyte can promote the curing of the system.

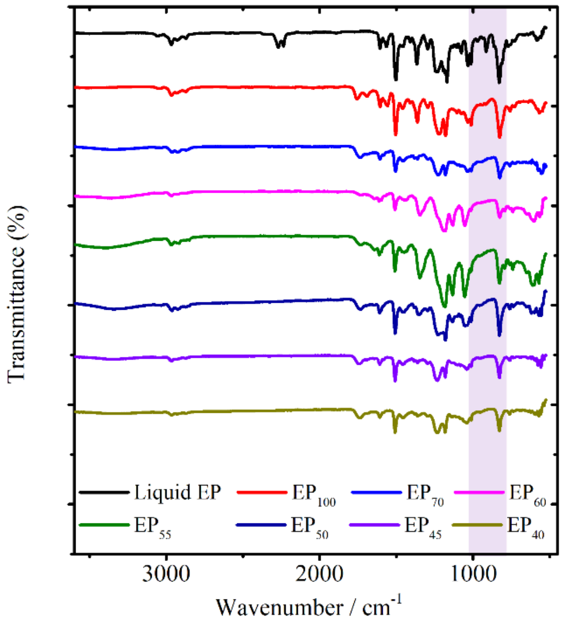

Supplementary Fig. 4 | FT-IR spectra of the uncured mixture of EP_{50} (liquid EP), EP_{100}, EP_{70}, EP_{60}, EP_{55}, EP_{50}, EP_{45}, EP_{40}.

In Supplementary Fig. 4, band at 1500 cm^{-1} is the characteristic peak of the benzene ring, while bands at 2270 cm^{-1} and 915 cm^{-1} is ascribed to the −OCN group and the epoxy group, respectively. After curing, the characteristic peaks of epoxy and −OCN groups disappeared, indicating the successfully crosslinking via the reaction between these two groups[6, 7]. In the IR spectrum of cured solid electrolyte, the band of the imidazole ring overlaps with the that of the benzene ring, and the new bands in 500 ~ 1000 cm^{-1} are assigned to the in-plane bending vibration of the C−F bond and C−H bond of EMIM-TFSI[7].

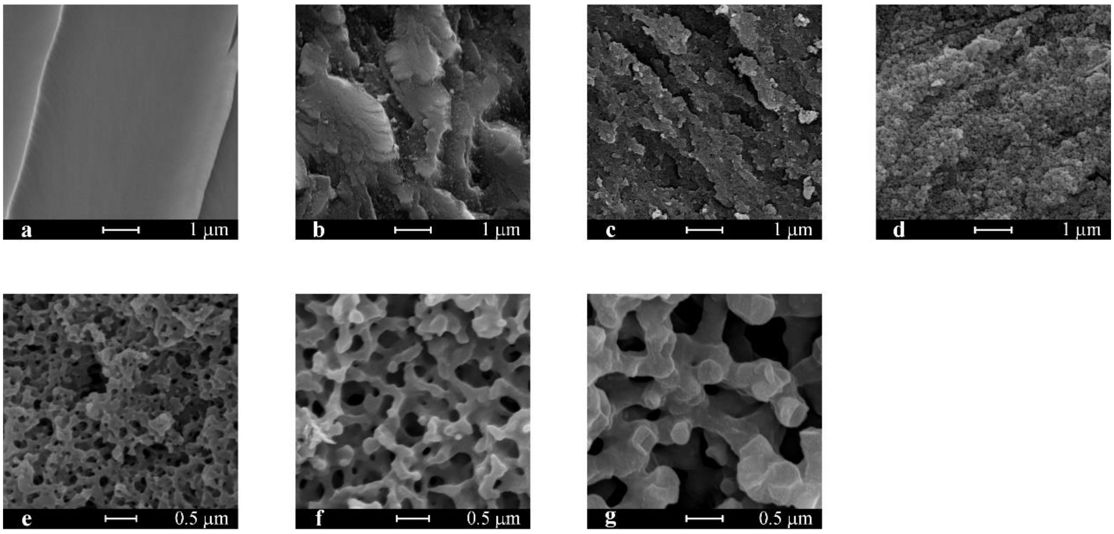

Supplementary Fig. 5 | a-g SEM images of EP_{100}, EP_{70}, EP_{60}, EP_{55}, EP_{50}, EP_{45}, EP_{40}.

A small amount of propylene carbonate (PC) was added into the LE to adjust the morphology of the solid electrolyte. According to the literature, PC can make the mixture of epoxy resin and LE more uniform and the pore size smaller[1, 8-10]. Moreover, with the increase of lithium ion concentration, PC can delay the process of curing phase separation[8].

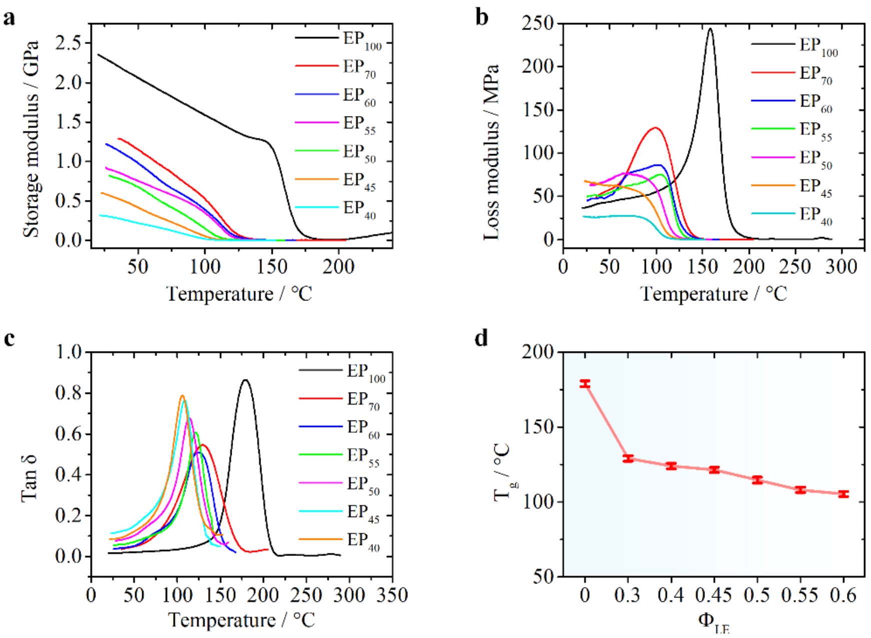

Supplementary Fig. 6 | Dynamic mechanical analysis (DMA) of different EPs. a Storage modulus curves. **b** Loss modulus curves. **c** The tan δ curves. **d** T_g of different solid electrolyte measured with DMA data.

In Supplementary Fig. 6, the storage modulus of solid electrolytes decreases drastically with the increase of temperature and is much lower than that of pure epoxy resin[11]. The presence of the IL between the molecular chains of the resin enhances the flexibility of the molecular chains, and the ability of the resin to resist deformation when subjected to external stress is reduced. The loss modulus gradually decreases with the increase of LE content. The peak value of the loss factor (tan δ) decreases first and then increases with the increase of the electrolyte content, and the position of the peak gradually moves to the lower temperature. When the addition amount of LE is less than 40%, the electrolyte improves the flexibility of the molecular chain, reduces the system viscosity and internal friction resistance, and reduces the peak loss factor. When the addition amount is higher than 40%, the system presents a dual continuous phase structure. Under the dual effects of temperature and stress,

friction occurs between the two phases, the internal friction resistance increases instead, and the peak loss factor increases. It further confirms that the whole system had obvious phase separation after adding LE, consistent with the SEM results[12-15]. The error bar is calculated from the standard deviation of results obtained in three different solid electrolytes.

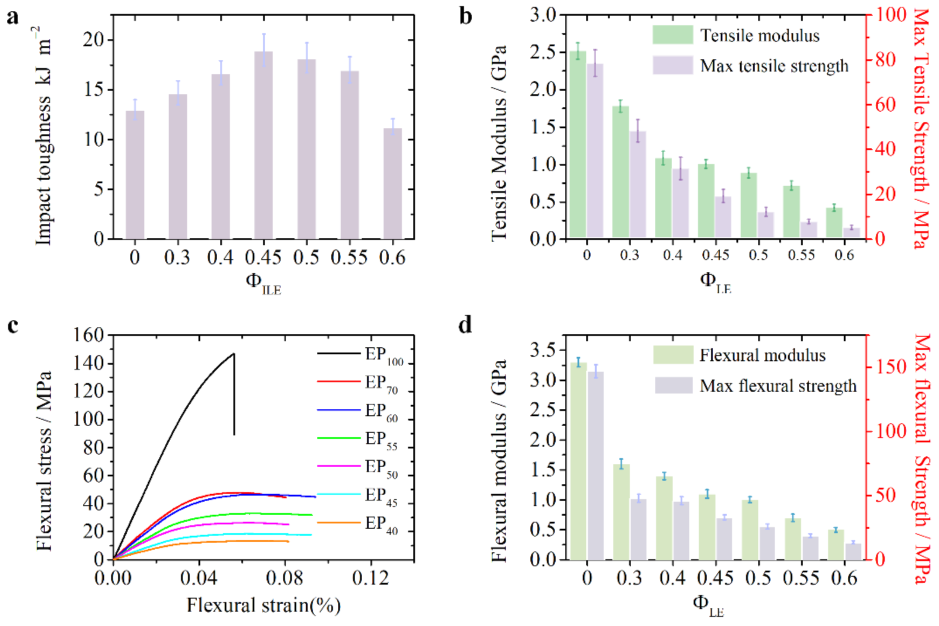

Supplementary Fig. 7 | a Impact strength graph of polymer electrolyte with different electrolyte content. **b** Tensile modulus and strength of solid electrolytes. **c** Flexural stress-strain curves of epoxy resin and different solid electrolytes. **d** Flexural modulus and strength of solid electrolytes.

As the electrolyte content increases, the impact strength of the solid electrolyte samples increases first and then decreases. In the solid electrolyte, the LE phase cannot bear the load, so the flexural strength of the system gradually decreases with the increase of LE content[16]. The IL acts as a plasticizer and exists between the resin molecular chain, and thus improves the impact strength of the system. The error bar is calculated from the standard deviation of results obtained in three different solid electrolytes.

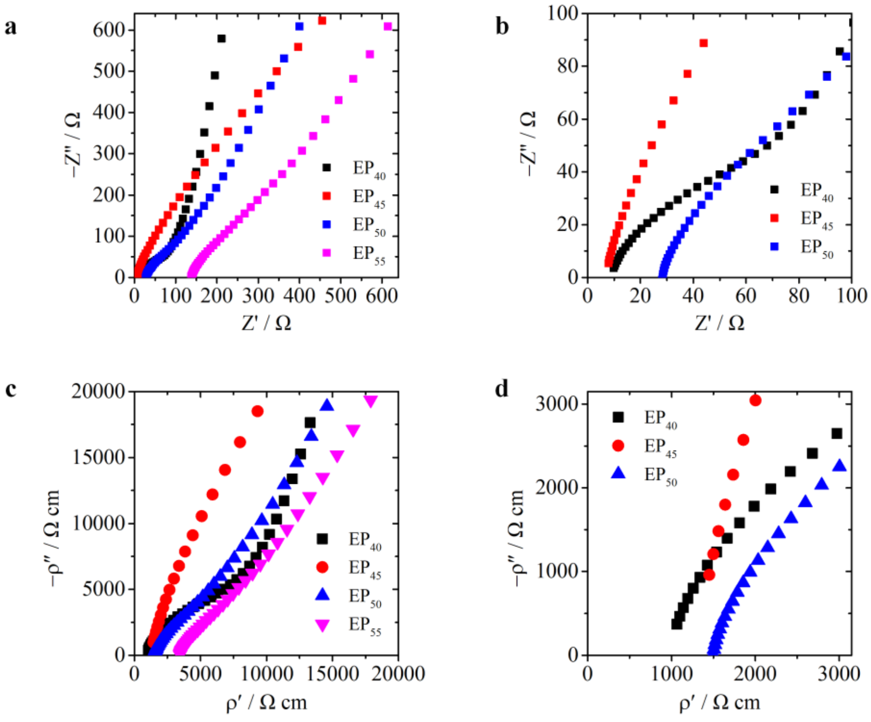

Supplementary Fig. 8 | a-b Enlarged view of impedance spectra of different solid electrolytes between block electrodes. **c-d** The formal resistivity (ρ) calculated from data in **(a)** and **(b)**. ($\rho = ZA/d$, where A and d are the area and thickness of the electrolyte sample.)

The value of the intercept between EP_{40} and the X axis is 9.9 Ω, the area is 15.2 cm^2, the thickness is 0.142 cm, the conductivity is 0.95 mS cm^{-1}, the value of the intercept between EP_{45} and the X axis is 7.9 Ω, and the area is 20.25 cm^2, the thickness is 0.136cm, the conductivity is 0.7 mS cm^{-1}, the value of the intercept between EP_{50} and the X-axis is 28.3 Ω, the area is 10.24 cm^2, the thickness is 0.188 cm, and the conductivity is 0.67 mS cm^{-1}.

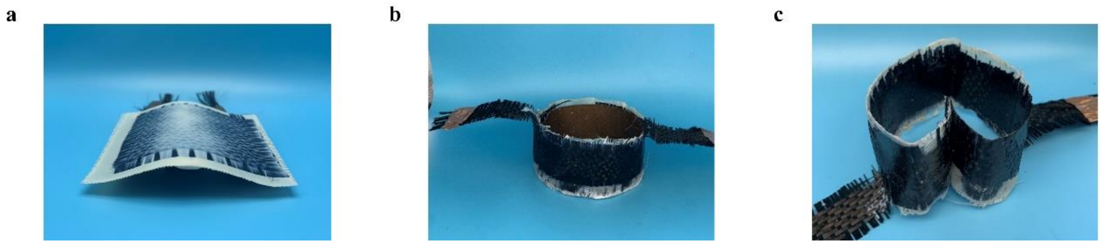

Supplementary Fig. 9 | a-c Photos of LEID-3 with different curved shapes.

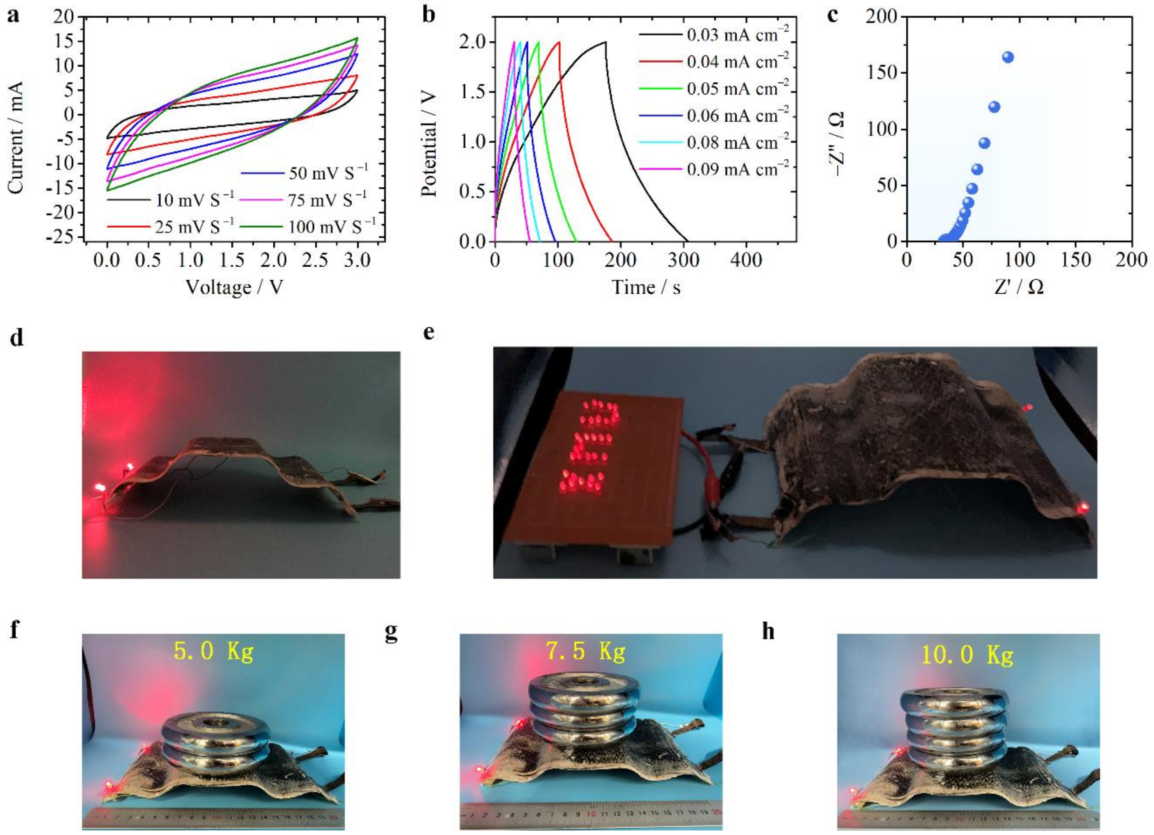

Supplementary Fig. 10 | a The CV distribution of automobile shell from 10 mV s^{-1} to 100 mV s^{-1}. **b** GCD test of Reduction parts of automobile shell by LEID-3 process. **c** impedance spectra of automobile shell. **d–h** The physical picture of the LED light can be lit without loading pressure and after loading pressure.

Supplementary Fig. 10 shows that the car shell model can work well as a power supply device and impact by heavy objects did not cause damage of the model and it has good fatigue resistance. Also see the Supplementary Movie 1.

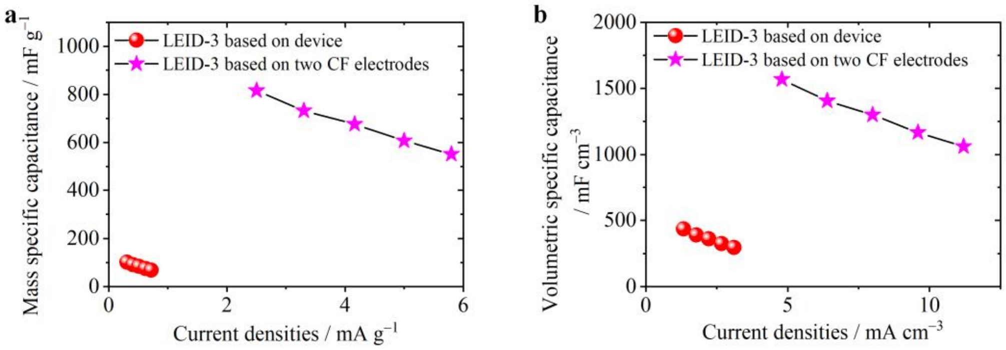

Supplementary Fig. 11 | a-b Specific capacitance of LEID-3. Based on device: the specific capacitance was calculated based on the mass or volume of the whole device, including the CF electrodes, GF separator, and solid electrolyte. Based on GF electrodes: the specific capacitance was calculated based on the mass or volume of the two GF electrodes.

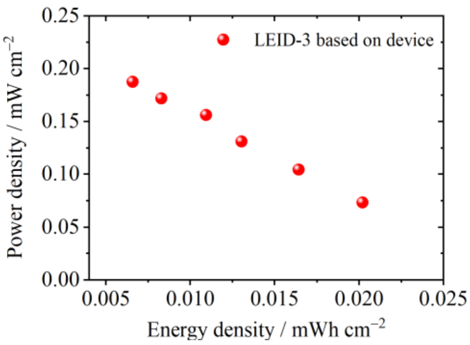

Supplementary Fig. 12 | Ragone plot of LEID-3 based on the areal of the whole device.

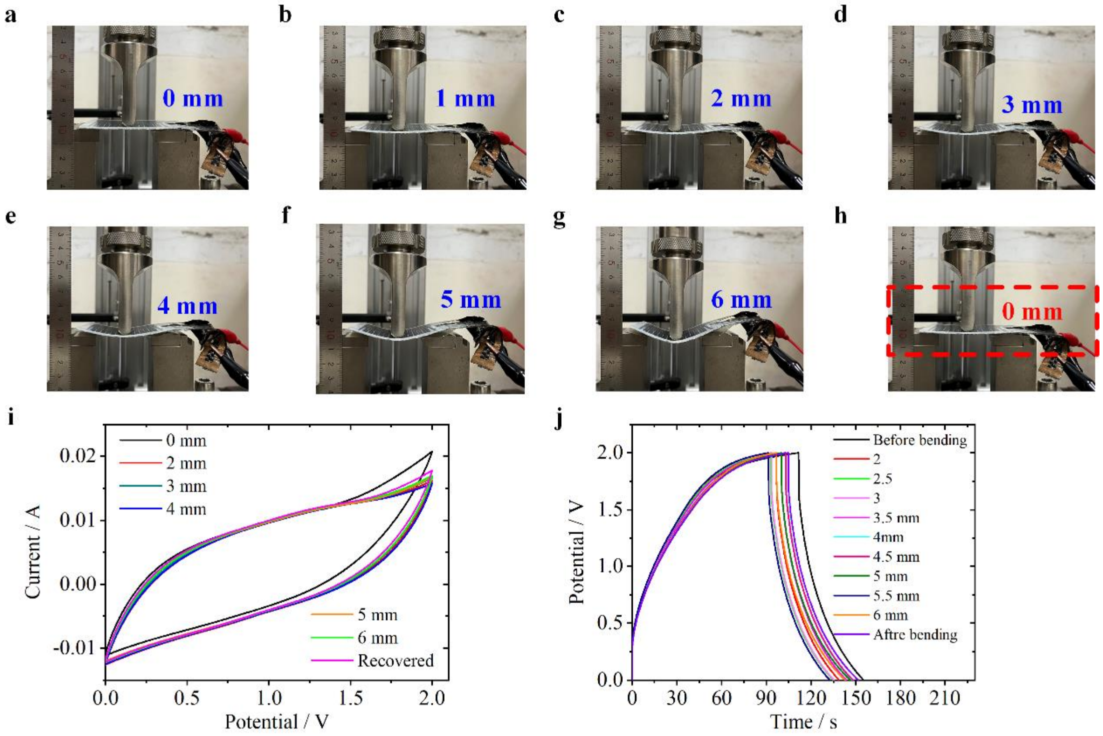

Supplementary Fig. 13 | a-h Physical photos of different bending deflections. **i-j** Electrochemical performance of the LEID-3 before and after bending.

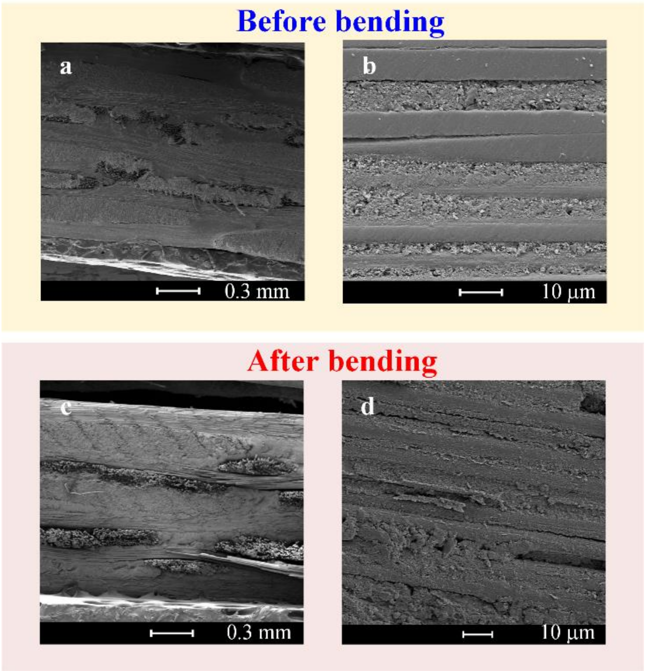

Supplementary Fig. 14 | SEM images of LEID-3 before bending (a, b) and after bending (c, d).

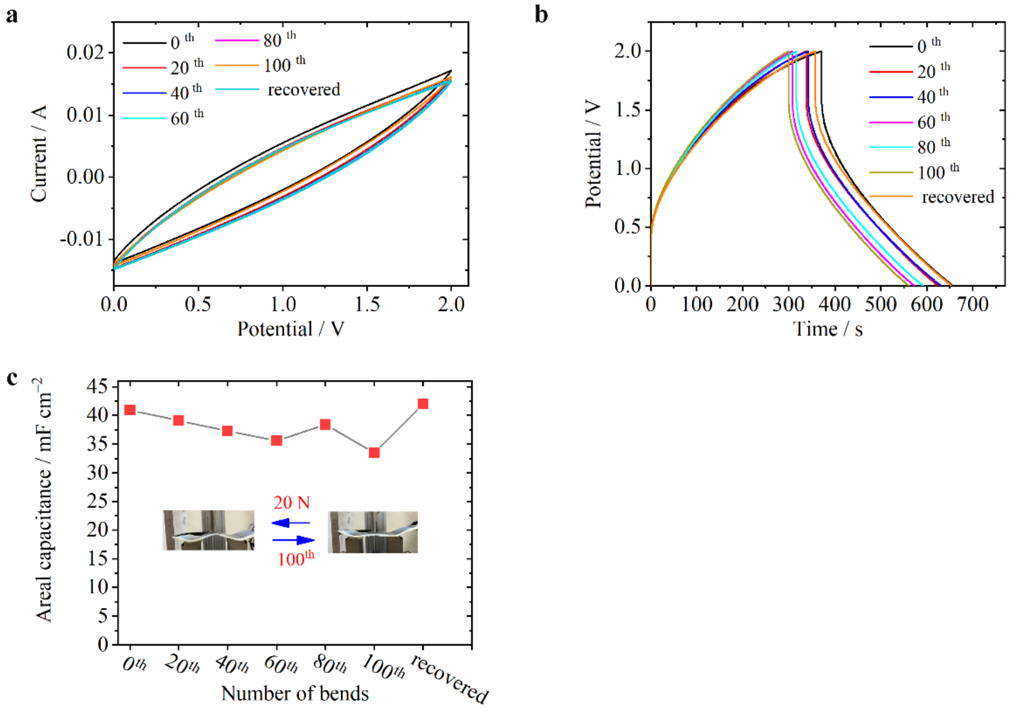

Supplementary Fig. 15 | Electrochemical performance of the LEID-3 after 100 bending/releasing cycles. **a** The CV curves. **b** The GCD curves. **c** Areal specific capacitance at different cycles.

The capacitance was measured under the bent state during the repeating bending, and under the original shape after 100 bending/releasing cycles. The specific capacitance under the bent state decreased gradually during continuous bending/releasing cycles but recovered to the original value immediately when the device shape was recovered.

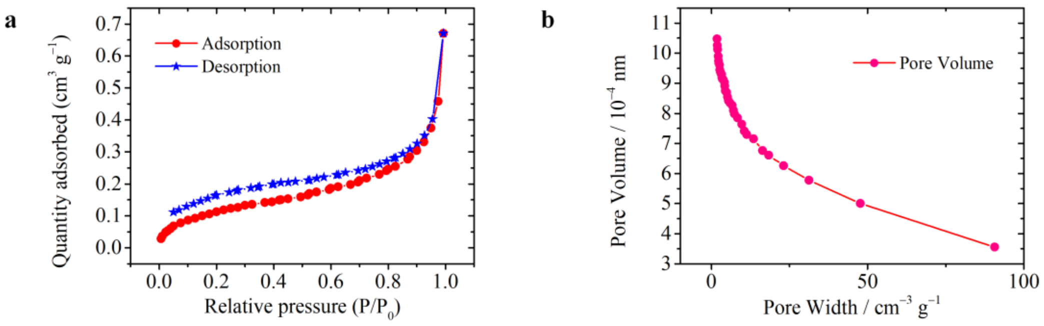

Supplementary Fig. 16 | BET test of the CF. **a** N_2 adsorption/desorption isotherms. **b** Corresponding pore size distributions.

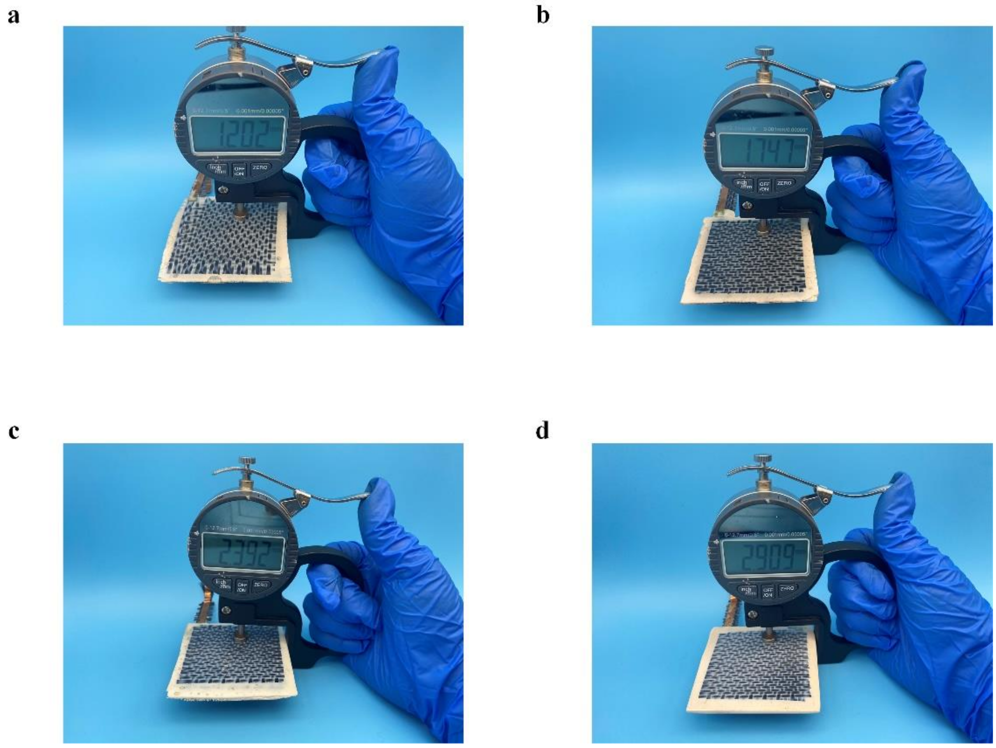

Supplementary Fig. 17 | a-d The photos of LEID-3, LEID-5, LEID-7, LEID-9.

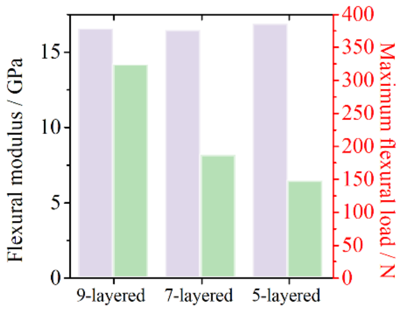

Supplementary Fig. 18 | Flexural modulus and maximum flexural load of LEIDs with different number of layers.

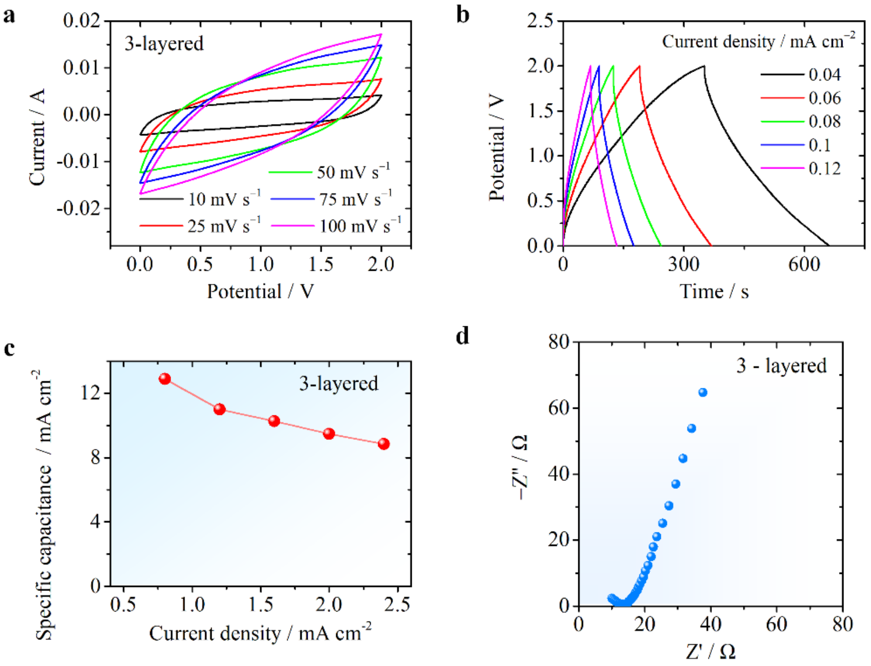

Supplementary Fig. 19 | Electrochemical properties of three-layered subdevice in LEID-7(I). a The CV curves. **b** The GCD curves. **c** Areal specific capacitance at different current densities. **d** Impedance spectra.

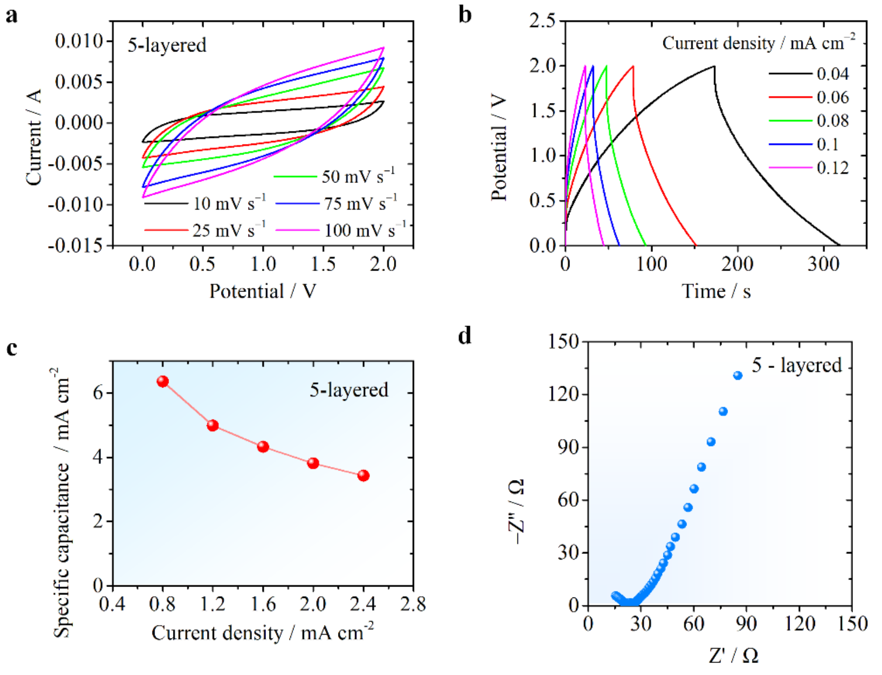

Supplementary Fig. 20 | Electrochemical properties of five-layered subdevice in LEID-7(I). a The CV curves. **b** The GCD curves. **c** Areal specific capacitance at different current densities. **d** Impedance spectra.

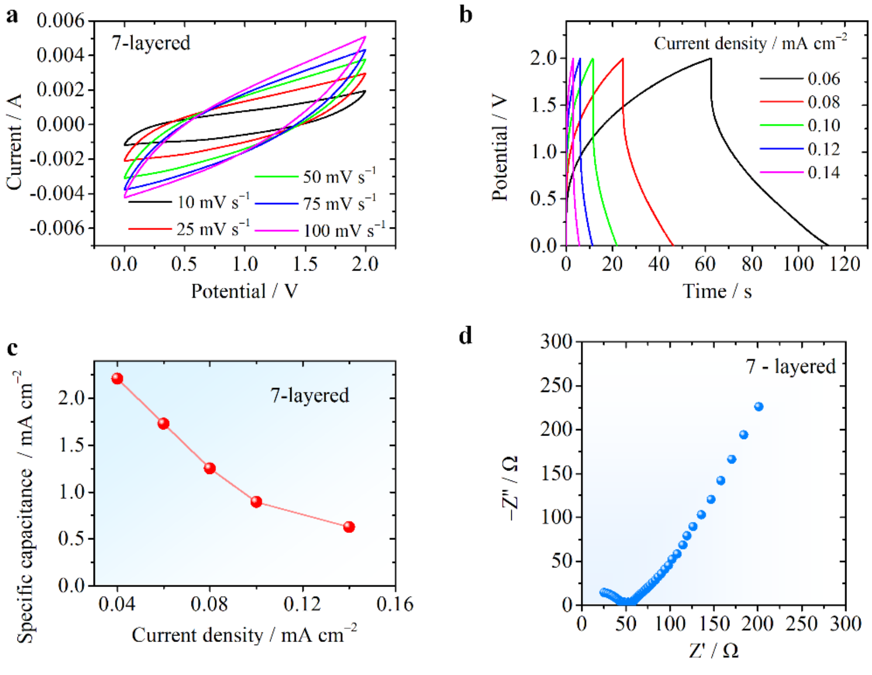

Supplementary Fig. 21 | Electrochemical properties of seven-layered subdevice in LEID-7(I). a The CV curves. **b** The GCD curves. **c** Areal specific capacitance at different current densities. **d** Impedance spectra.

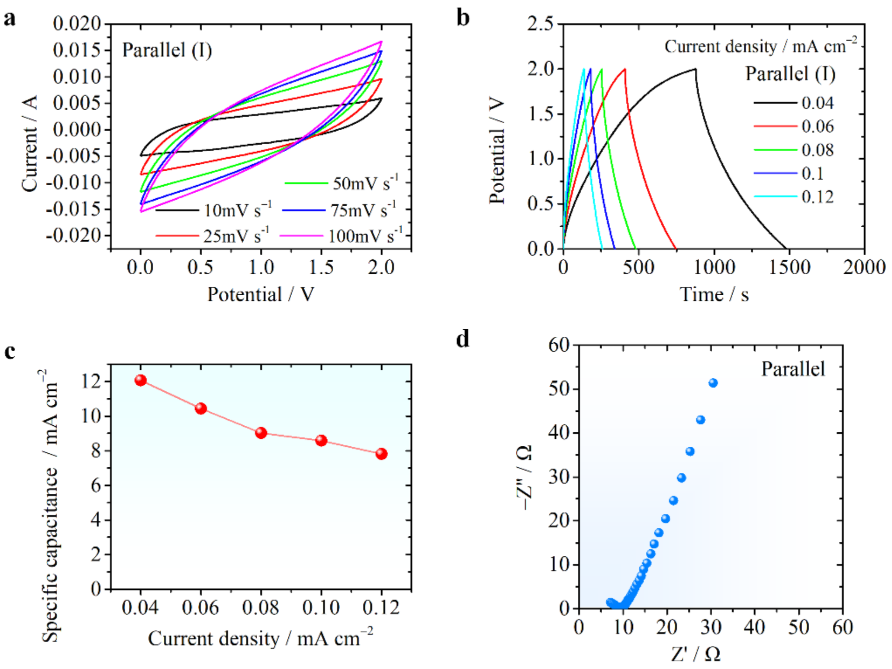

Supplementary Fig. 22 | Electrochemical properties of LEID-7(I) in parallel mode. a The CV curves. **b** The GCD curves. **c** Areal specific capacitance at different current densities. **d** Impedance spectra.

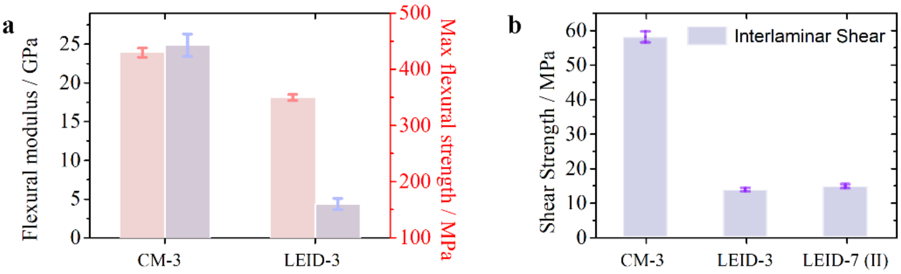

Supplementary Fig. 23 | a Bending stress-strain curves of CM-3 and LEID-3. **b** Interlaminar shear strength of CM-3, LEID-3, and LEID-7(II).

The error bar is calculated from the standard deviation of results obtained in three different devices.

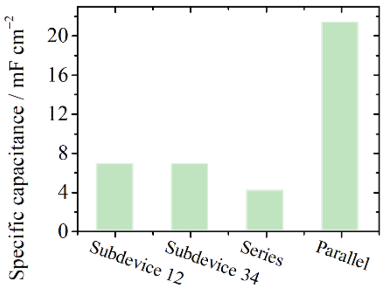

Supplementary Fig. 24 | Areal specific capacitance of LEID-7(II). and its subdevice in different modes.

Supplementary Table 1 | The composition of the precursor mixture for different electrolytes.

Sample	EP5284 : EMIm-TFSI : LiTFSI : PC (by weight)
EP_{100}	100 : 0 : 0 : 0.
EP_{70}	70 : 30 : 13 : 0.3
EP_{60}	60 : 40 : 17.4 : 0.4
EP_{55}	55 : 45 : 19.5 : 0.45
EP_{50}	50 : 50 : 21.8 : 0.5
EP_{45}	45 : 55 : 23.9 : 0.55
EP_{40}	40 : 60 : 26.1 : 0.6

Supplementary Table 2 | Conductivity comparison of ionic liquid electrolytes and organic electrolytes.

Electrolyte sample	Ionic conductivity / mS cm^{-1}
Ethyl methyl carbonate (EMC) / 1 M LiTFSI	3.4
Dimethyl carbonate (DMC) / 1 M LiTFSI	4.1
Diethyl carbonate (DEC) / 1 M LiTFSI	2.5
Propylene carbonate (PC) / 1 M LiTFSI	5.5
EMIm-TFSI / 1 M LiTFSI	2.9
EMIm-TFSI / 2.3 M LiTFSI	2.1
EC : DMC =1 : 1 vol% / 1 M $LiPF_6$	7.9

**Supplementary Table 3 | **The Ionic conductivity and mechanical strength comparison of the solid electrolytes in literature with EP_{50} in this work.

Electrolyte	Ionic conductivity / mS cm^{-1}	Young's modulus / GPa
MVR444 / EMIm-TFSI / LiTFSI[17]	0.007	0.81
Epoxy / SN / LiTFSI[5]	0.35	0.3
CCR Epoxy / $BMIBF_4$ / $LiBF_4$[18]	0.00135	0.045
Epoxy / PEG / LiTF[19]	0.0086	0.135
DGEBA Epoxy / SN / LiTFSI[20]	0.2	0.001
DGEBA Epoxy / BMIm-TFSI/ LiTFSI[4]	0.26	0.5
EP_{50} in this work	0.67	1

Supplementary Table 4 | Sample thickness and composition of LEIDs.

Structure of LEID	Epoxy resin/electrolyte mass ratio	Thickness (mm)	Mean fiber volume fraction (%)	Resin volume fraction (%)	ILS volume fraction (%)
$(CF/GF/CF)_{EP50}$	50/50	1.20 ± 0.01	41.7%	29.2%	29.1%
$(CF/GF/CFGF/CF)_{EP50}$	50/50	1.74 ± 0.01	42.5%	28.8%	28.7%
$(CF/GF/CF/GF/CF/GF/CF)_{EP50}$	50/50	2.39 ± 0.01	40.2%	30.0%	29.8%
$(CF/GF/CF/GF/CF/GF/CF/GF/CF)_{EP50}$	50/50	2.91 ± 0.01	40.4%	29.9%	29.7%
$(CF/GF/CF)_{EP50}/GF_{EP70}/(CF/GF/CF)_{EP50}$	50/50, 70/30	2.25 ± 0.01	38.6%	32.8%	28.6%

**Supplementary Table 5 | ** Comparison of the specific capacitance of LEID in this work with other solid-state supercapacitors in the literature [2, 11, 15, 21-27].

Electrode	Separator	Electrolyte	Specific capacitance	Reference
CF	GF	EP-IL	C_a=32.4 mF cm^{-2} [b], C_v=1297.8 mF cm^{-3} [b], C_g=675.9 mF g^{-1} [b]	This work
MnO_2-CF	GF	EP-IL	C_a=5.68 mF cm^{-2} [b], C_v=82 mF cm^{-3} [b], C_g=49 mF g^{-1} [c]	22
Vertical Graphene/MnO_2-CF	GF	EP-IL	C_v=30.7 mF cm^{-2} [c]	21
CuO-CF	GF	Polyester -LiTf-$EMIMBF_4$	C_g=6.75 F g^{-1} [a]	15
Activated CF	FP	EP-$TEABF_4$	C_g=25.4 mF g^{-1} [c]	23
Carbon aerogel-CF	GF	PEGDGE-IL	C_a=3.15 mF cm^{-2} [b], C_v=34.6 mF cm^{-3} [b], C_g=71.2 mF g^{-1} [b]	2
Graphene nanoplatelet-CF	FP	DGEBA-$LiClO_4$	C_v=118.7 mF cm^{-3} [a]	24
Urea-Activated GO-CF	GF	PEGDGE-IL	C_v=82.3 mF cm^{-3} [b]	25
ZnO-CF	GF	Polyester -LiTf-$EMIMBF_4$	C_g=10.6 F g^{-1} [a]	11
Cu-Co-Se-CF	KF	Polyester -LiTf-$EMIMBF_4$	C_g=28.6 F g^{-1} [a]	26
MWCNTs-CF	GF	PEG-LiTf	C_g=125 mF g^{-1} [c]	27

[a] Specific capacitance was calculated based on the mass of active materials. (witout the mass of CF and electrolyte)

[b] Specific capacitance was calculated based on the mass of electrodes. (The mass of CF or total mass of active materials and CF)

[c] Specific capacitance was calculated based on the mass of device.

References

1. Shirshova, N. *et al.* Structural supercapacitor electrolytes based on bicontinuous ionic liquid-epoxy resin systems. *J. Mater. Chem. A* **1**, 15300-15309 (2013).

2. Qian, H., Kucernak, A.R., Greenhalgh, E.S., Bismarck, A. & Shaffer, M.S.P. Multifunctional structural supercapacitor composites based on carbon aerogel modified high performance carbon fiber fabric. *ACS Appl. Mater. Interfaces* **5**, 6113-6122 (2013).

3. Javaid, A., Ho, K.K.C., Bismarck, A., Shaffer, M.S.P., Steinke, J.H.G. & Greenhalgh, E.S. Multifunctional structural supercapacitors for electrical energy storage applications. *J. Compos. Mater.* **48**, 1409-1416 (2013).

4. Kwon, S.J., Kim, T., Jung, B.M., Lee, S.B. & Choi, U.H. Multifunctional epoxy-based solid polymer electrolytes for solid-state supercapacitors. *ACS Appl. Mater. Interfaces* **10**, 35108-35117 (2018).

5. Chopade, S.A., Au, J.G., Li, Z., Schmidt, P.W., Hillmyer, M.A. & Lodge, T.P. Robust polymer electrolyte membranes with high ambient-temperature lithium-ion conductivity via polymerization-induced microphase separation. *ACS Appl. Mater. Interfaces* **9**, 14561-14565 (2017).

6. Kim, B.S. Effect of cyanate ester on the cure behavior and thermal stability of epoxy resin. *J. Appl. Polym. Sci.* **65**, 85-90 (1997).

7. Li, C.P. & Chuang, C.M. Thermal and dielectric properties of cyanate ester cured main chain rigid-rod epoxy resin. *Polymers* **13**, 2917 (2021).

8. Shirshova, N. *et al.* Composition as a means to control morphology and properties of epoxy based dual-phase structural electrolytes. *J. Phys. Chem. C* **118**, 28377-28387 (2014).

9. Yu, Y. *et al.* Co-continuous structural electrolytes based on ionic liquid, epoxy resin and organoclay: Effects of organoclay content. *Mater. Des.* **104**, 126-133 (2016).

10. Yu, Y. *et al.* Multifunctional structural lithium ion batteries based on carbon fiber reinforced plastic composites. *Compos. Sci. Technol.* **147**, 62-70 (2017).

11. Deka, B.K., Hazarika, A., Kwon, O., Kim, D., Park, Y.-B. & Park, H.W. Multifunctional enhancement of woven carbon fiber/zno nanotube-based structural supercapacitor and polyester resin-domain solid-polymer electrolytes. *Chem. Eng. J.* **325**, 672-680 (2017).

12. Pereira, T., Guo, Z., Nieh, S., Arias, J. & Hahn, H.T. Embedding thin-film lithium energy cells in structural composites. *Compos. Sci. Technol.* **68**, 1935-1941 (2008).

13. Shirshova, N. *et al.* Multifunctional structural energy storage composite supercapacitors. *Faraday Discuss.* **172**, 81-103 (2014).

14. Shirshova, N. *et al.* Structural composite supercapacitors. *Compos. Pt. A-Appl. Sci. Manuf.* **46**, 96-107 (2013).

15. Deka, B.K., Hazarika, A., Kim, J., Park, Y.-B. & Park, H.W. Multifunctional cuo nanowire embodied structural supercapacitor based on woven carbon fiber/ionic liquid-polyester resin. *Compos. Pt. A-Appl. Sci. Manuf.* **87**, 256-262 (2016).

16. Munoz, B.K. *et al.* Epoxy resin systems modified with ionic liquids and ceramic nanoparticles as structural composites for multifunctional applications. *Polymer* **214**, (2021).

17. Shirshova, N. *et al.* Structural supercapacitor electrolytes based on bicontinuous ionic liquid–epoxy resin systems. *J. Mater. Chem. A* **1**, 15300 (2013).

18. Westover, A.S. *et al.* Multifunctional high strength and high energy epoxy composite structural supercapacitors with wet-dry operational stability. *J. Mater. Chem. A* **3**, 20097-20102 (2015).

19. Feng, Q. *et al.* The ionic conductivity, mechanical performance and morphology of two-phase structural electrolytes based on polyethylene glycol, epoxy resin and nano-silica. *Mat. Sci. and Eng.: B* **219**, 37-44 (2017).

20. Jang, H.K., Jung, B.M., Choi, U.H. & Lee, S.B. Ion conduction and viscoelastic response of epoxy-based solid polymer electrolytes containing solvating plastic crystal plasticizer. *Macromol. Chem. Phys.* **219**, 1700514 (2018).

21. Huang, F. *et al.* Surface functionalization of electrodes and synthesis of dual-phase solid electrolytes for structural supercapacitors. *ACS Appl. Mater. Interfaces* **14**, 30857-30871 (2022).

22. Sha, Z. *et al.* Synergies of vertical graphene and manganese dioxide in enhancing the energy density of carbon fibre-based structural supercapacitors. *Compos. Sci. Technol.* **201**, 10856 (2021).

23. Reece, R., Lekakou, C. & Smith, P.A. A structural supercapacitor based on activated carbon fabric and a solid electrolyte. *Mater. Sci. Technol.* **35**, 368-375 (2019).

24. Javaid, A., Zafrullah, M.B., Khan, F.u.H. & Bhatti, G.M. Improving the multifunctionality of structural supercapacitors by interleaving graphene nanoplatelets between carbon fibers and solid polymer electrolyte. *J. Compos. Mater.* **53**, 1401-1409 (2019).

25. Ganguly, A., Karakassides, A., Benson, J., Hussain, S. & Papakonstantinou, P. Multifunctional structural supercapacitor based on urea-activated graphene nanoflakes directly grown on carbon fiber electrodes. *ACS Appl. Energy Mater.* **3**, 4245-4254 (2020).

26. Deka, B.K. *et al.* Bimetallic copper cobalt selenide nanowire-anchored woven carbon fiber-based structural supercapacitors. *Chem. Eng. J.* **355**, 551-559 (2019).

27. Hudak, N.S., Schlichting, A.D. & Eisenbeiser, K. Structural supercapacitors with enhanced performance using carbon nanotubes and polyaniline. *J. Electrochem. Soc.* **164**, A691-A700 (2017).
