# Supplementary material for: High-strength and machinable load-bearing integrated electrochemical capacitors based on polymeric solid electrolyte
Source: Nat Commun. 2023 Jan 4;14:64. doi: 10.1038/s41467-022-35737-w (PMC9812976; doi:10.1038/s41467-022-35737-w)
Supplement: Supplementary file 3 — Description of Additional Supplementary Files [file 41467_2022_35737_MOESM3_ESM.pdf]

Description of Additional Supplementary Files

File Name: Supplementary Movie 1
Description: A LEID-3 car shell model lighting up light-emitting diodes during the impact of the ten-kilogram weight.

File Name: Supplementary Movie 2
Description: LEID-3 powering a small fan after being drilled three holes.

File Name: Supplementary Movie 3
Description: A running car model assembled by LEID-3 and plastic screws.
